# Regulation of Dopamine D1 Receptor Dynamics within the Postsynaptic Density of Hippocampal Glutamate Synapses

**DOI:** 10.1371/journal.pone.0074512

**Published:** 2013-09-06

**Authors:** Laurent Ladepeche, Luting Yang, Delphine Bouchet, Laurent Groc

**Affiliations:** 1 Univ. de Bordeaux, Interdisciplinary Institute for Neuroscience, Unité Mixte de recherche UMR 5297, Bordeaux, France; 2 Centre National de la Recherche Scientifique CNRS, IINS UMR 5297, Bordeaux, France; Baylor College of Medicine, United States of America

## Abstract

Dopamine receptor potently modulates glutamate signalling, synaptic plasticity and neuronal network adaptations in various pathophysiological processes. Although key intracellular signalling cascades have been identified, the cellular mechanism by which dopamine and glutamate receptor-mediated signalling interplay at glutamate synapse remain poorly understood. Among the cellular mechanisms proposed to aggregate D1R in glutamate synapses, the direct interaction between D1R and the scaffold protein PSD95 or the direct interaction with the glutamate NMDA receptor (NMDAR) have been proposed. To tackle this question we here used high-resolution single nanoparticle imaging since it provides a powerful way to investigate at the sub-micron resolution the dynamic interaction between these partners in live synapses. We demonstrate in hippocampal neuronal networks that dopamine D1 receptors (D1R) laterally diffuse within glutamate synapses, in which their diffusion is reduced. Disrupting the interaction between D1R and PSD95, through genetical manipulation and competing peptide, did not affect D1R dynamics in glutamatergic synapses. However, preventing the physical interaction between D1R and the GluN1 subunit of NMDAR abolished the synaptic stabilization of diffusing D1R. Together, these data provide direct evidence that the interaction between D1R and NMDAR in synapses participate in the building of the dopamine-receptor-mediated signalling, and most likely to the glutamate-dopamine cross-talk.

## Introduction

Dopamine, one of the major brain neuromodulator, regulates several physiological functions such as motion, motivation, novelty, reward, memory, and a dysregulation of the dopaminergic signalling is central in pathological conditions such as during Parkinson’s disease and schizophrenia [1]–[5]. Dopamine receptors are schematically divided in two classes, characterized by their G protein-coupled effector molecules [6]. In the hippocampus and cortex, pyramidal neurons express dopamine D1 and D5 receptors (D1/5R family) along their dendritic tree and in the close vicinity of the excitatory glutamate synapses [7]–[9]. These receptors are positively coupled to production of cyclic adenosine monophosphatase (cAMP) through adenylyl cyclase with the well-characterized downstream effectors: protein kinase A (PKA), cAMP-responsive element binding protein (CREB), and DARPP-32 [10], [11]. Schematically, once dopamine is released in the hippocampus it leads at the postsynaptic level to i) an activation of D1/5R, PKA and downstream signalling, ii) an increase in protein synthesis, iii) an increase in surface GluA1-AMPA and GluN1-NMDA receptors, and iv) a modulation of NMDA receptor (NMDAR)-dependent synaptic plasticity [12]–[18].

The functional relationship between dopamine receptor- and NMDAR-mediated signalling has thus been under high scrutiny. Most of the studies have focused their attention on the intracellular pathways, identifying for instance the calcium-dependent PKA/DARPP-32 signalling cascade as an important intracellular mediator of the cross-talk [19]–[27]. In addition, D1R and NMDAR directly interact [28], [29] and such protein-protein interaction also regulates NMDAR-mediated signalling and working memory [30], [31]. Finally, the membrane-associated guanylate kinase (MAGUK) proteins, such as postsynaptic density 95 (PSD95), organize NMDAR synaptic distribution and consequently regulate the strength and plasticity of synapses [32]. Of interest, PSD95 directly interacts with D1R, regulating its trafficking and function [11], [33]–[35]. Thus, the functional interplay between dopamine D1R and NMDAR can take place at several cellular loci, i.e. intracellular mediators, direct receptor interactions, indirect receptor interaction through PSD95.

At the plasma membrane level, ionotropic and metabotropic receptors laterally diffuse, explore rather large area in dendrite, and constantly exchange between synaptic areas and extrasynaptic compartment [36], [37]. This process applies to the NMDAR that diffuse at the surface of hippocampal neurons in a receptor composition-, age-, and activity-dependent manner [38]–[40]. In glutamate synapses, diffusing NMDAR are actively anchored by MAGUK proteins, such as PSD95 [41]. D1R have also been shown to diffuse at the neuronal surface and interaction with intracellular partners (e.g. PSD95) has been proposed to regulate their membrane behaviours [42], [43]. Thus, the functional crosstalk between the glutamatergic NMDAR and dopamine D1R signalling likely involve plasma membrane interplay. In glutamate synapses, D1R content could be regulated either by membrane direct protein interaction with NMDAR or by indirect regulation through common interactor such as PSD95. In the present report, we directly address this question by investigating at the single nanoparticle level [37] the molecular mechanism by which D1R are trapped in glutamate synapse area.

## Materials and Methods

### Primary Cell Culture, Protein Expression and Synaptic Live Staining

Cultures of hippocampal neurons were prepared from E18 Sprague-Dawley rats. All experiments were conducted in strict compliance with European Communities Council and French Directives for care of laboratory animals European directives and French laws on animal experimentation (approved by Bordeaux University Institutional Animal Care and ethics committee; LG authorization # 3306009). All efforts have been made to use the minimum number of animals necessary to perform statistically valid analysis, and to reduce animal suffering. The pregnant rat were sacrifice by cervical dislocation after anesthesia. Briefly, cells were plated at a density of 60×10^3^ cells per ml on poly-lysine pre-coated coverslips and kept at 37°C in 5% CO_2_. After 4 days *in vitro* (div), the original plating neurobasal culture medium (Invitrogen) complemented with horse-serum was replaced with a serum-freemedium. For D1R-CFP receptor expression, 7–10 div hippocampal cultured neurons were transfected 24–72 h before each experiment using the Effectene reagent (Qiagen). For synaptic staining, neurons were co-transfected with the postsynaptic marker PSD95 fused to the GFP on its N-terminus (PSD95-NT_GFP_) or C-terminus (PSD95-CT_GFP_) part depending on the experiment. Schematically, 2 µg of DNA were mixed with 25 µl of Effectene and 8 µl of Enhancer in 150 µl of reaction buffer, and then added the mixture to cultured neurons which were transferred to serum-free neurobasal medium 10 min beforehand. After an incubation period of 45 min, neurons were placed in the old medium again.

### Cell Surface Delivery Assay

HEK 293 cells were cotransfected in 12-well tissue culture plates with D1R-CFP and PSD95-CT_GFP_ or PSD95-NT_GFP_ (1.5 µg of total DNA per well). For immunostaining, live cells were labelled post-transfection (24–36 h) using a monoclonal anti-GFP antibody (Roche, 1∶10000, 15 min, 37°C) in culture medium in order to detect surface D1R. Then, cells were fixed with 4% paraformaldehyde/4% sucrose for 15 min, washed and incubated with secondary antibodies anti-mouse Alexa568 antibodies (Molecular Probes; 1∶1000, 45 min, RT). Cells were washed, mounted, and preparations were kept at 4°C until quantification. The fluorescence was analyzed using imaging tools from Metamorph software (Universal Imaging Corporation, PA, USA).

### Peptide Incubation

Hippocampal neurons were incubated with TAT peptide for 10 minutes at the final concentration of 10 µM. The TAT-t_2_ peptide contains the D1R C-tail from L_387_ to L_416_ and the TAT-PSD-D1 peptide contains the D1R C-tail from L_401_ to T_446_ and their respective control non-sense peptides consist in a scramble sequence of the same amino acids. Both peptides are cell permeant by containing a TAT sequence (GRKKRRQRRR). SKF38393 was purchased from Sigma-Aldrich and was made up as a 10 mM stock solution.

### Single Quantum Dot Tracking and Surface Diffusion Calculation

Quantum dots (QD) 655 Goat F(ab’)2 anti-mouse IgG (Invitrogen) were first incubated for 30 min with the monoclonal anti-GFP (Roche, 1 µg) antibody. Non-specific binding was blocked by additional casein (Vector Laboratories, USA) to the QD 15 min before use. Neurons were first incubated for 10 min at 37°C in culture medium with pre-coated QDs (final dilution 1∶20000 for anti-GFP coupled QDs). For the specific experiments, TAT peptides were applied together with the pre-coated QDs. Detection of the QDs was performed by using a mercury lamp and appropriate excitation/emission filters. Images were obtained with an integration time of 50 ms with up to 1000 consecutive frames. Signals were detected using a EMCCD camera (Quantem, Roper Scientific). QDs were followed on randomly selected dendritic regions for up to 30 min. QD recording sessions were processed with the Metamorph software (Universal Imaging Corporation, PA, USA). The instantaneous diffusion coefficient, D, was calculated for each trajectory, from linear fits of the first 4 points of the mean-square-displacement versus time function using:
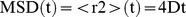



The two-dimensional trajectories of single molecules in the plane of focus were constructed by correlation analysis between consecutive images using a Vogel algorithm.

### Statistical Analysis

The instantaneous diffusion coefficient is reported as the median ± 25–75% (IQR). The other data are expressed as mean ± sem. Comparisons between groups for instantaneous diffusion coefficients were performed using Mann Whitney test (pair comparison) or Kruskal-Wallis followed by Dunn’s Multiple Comparison Test (group comparison). All the other comparisons between groups were performed using parametric statistical tests, Student-t test (pair comparison), ANOVA followed by Newman-Keuls Multiple Comparison Test (group comparison), or Kolmogorov-Smirnov test (distribution comparison). Significance levels were defined as *p<0.05, **p<0.01, ***p<0.001.

## Results

### D1R Surface Dynamics in Native PSD95-containing Synaptic Structures

**Figure 1:**
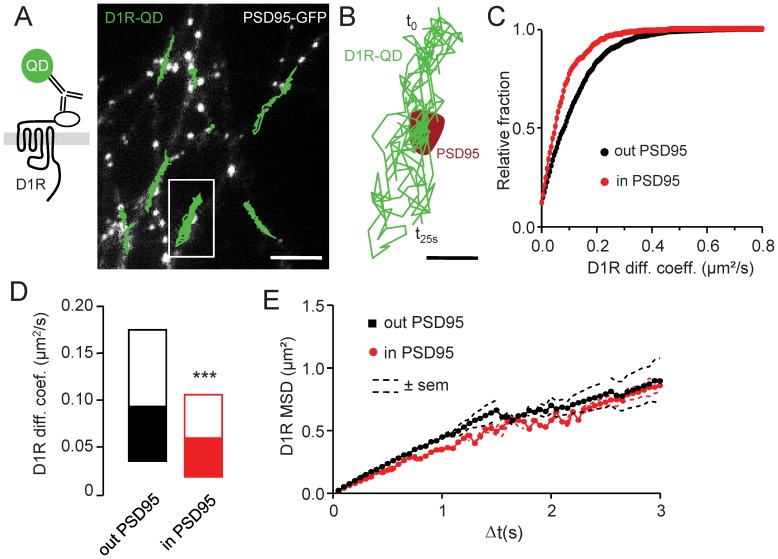
D1R surface dynamics in native PSD95-containing synaptic structures. (**A**) Surface D1R-CFP were labeled with a single Quantum Dot (QD)-antibody complex, allowing single particle tracking (*left panel*). Representative trajectories (500 frames duration; 20 Hz acquisition rate) of multiple single surface D1R (green traces) recorded on a PSD95-GFP expressing hippocampal neuron (*right panel*, scale bar = 5 µm). (**B**) Representative single D1R trajectory crossing a PSD95-GFP cluster (red area). This is a magnification of the white line squared region of the left picture (scale bar = 1 µm). (**C**) Frequency distribution of the instantaneous diffusion coefficients of surface D1R either inside or outside of PSD95-GFP clusters. Note the shifted distribution toward lowerD1R diffusion coefficients inside synaptic areas when compared to those located in the extrasynaptic membrane (n = 1784 and 632 trajectories, respectively). (**D**) Distribution (median±25–75% range) of the instantaneous diffusion coefficients of surface D1R either inside or outside of PSD95-GFP clusters. The D1R surface diffusion is significantly reduced inside postsynaptic areas, i.e. PSD95 clusters, when compared to those located in the extrasynaptic membrane (n = 1784 and 632 trajectories respectively; P<0.001). (**E**) Plot of the mean square displacement (MSD, expressed in µm^2^) versus time of surface D1R trajectories either inside or outside of PSD95-GFP clusters (n = 1784 and 632 trajectories respectively). Note that synaptic and extrasynaptic D1R have similar diffusion behavior without increased confinement inside synapses.

D1R have been previously detected in the close vicinity and inside glutamate synapses of hippocampal and cortical pyramidal neurons [1], [2], [3]. In addition, these receptors laterally diffuse at the surface of striatal neurons and their lateral diffusion is altered in dendritic spines [42], [43], suggesting the presence of regulatory mechanisms inside synaptic areas. To shed light on the cellular mechanism that anchor D1R in synapses of hippocampal neurons, and in particular to investigate whether an active trapping of diffusing D1R is engaged in this process as described for other receptors (e.g. NMDAR, [41]), we tracked single D1R-QD at the surface of hippocampal neurons transfected with D1R containing at their N-terminus part a cyan fluorescent protein (D1R-CFP) (Fig. 1A). The postsynaptic location was determined by the co-transfection of hippocampal neurons with a PSD95 fused to the green fluorescent protein (PSD95-GFP). We first report that D1R diffuse at the surface of hippocampal neurons and explore large areas of the dendritic tree, confirming previous observations in striatal neurons [42], [43]. Within the postsynaptic density (PSD) area D1R surface dynamics slowed down (Fig. 1B), suggesting the presence of mechanism that interferes with D1R surface dynamics. The distributions of D1R diffusion coefficient measured outside and inside PSD95 clusters were compared. D1R diffusion was significantly lower inside PSD95 clusters as indicated by the left shift of the distribution inside PSD95 (Fig. 1C). Consistently, D1R diffusion coefficient median inside PSD95 clusters was significantly reduced when compared to the one outside clusters (Fig. 1D). These data suggest thus the existence of a regulatory mechanism that controls the synaptic retention of D1R, consistent with the active synaptic trapping described for other transmembrane receptors. As an example, NMDAR directly and physically binds to PDZ domain proteins such as PSD95 and this interaction regulates their active retention in synapse [41]. To further explore the behavior of D1R within PSD, we examined the mean square displacement (MSD) over time, which is an index of the area explored by the QD-receptor complexes. In synapses, this measurement provided a powerful way to detect the high confinement of receptors that result from their trapping by scaffold proteins [41], [44]–[47]. Quite surprisingly, there was no difference in the D1R MSD when compared between synaptic and non-synaptic compartment (Fig. 1E). The MSD exhibited a non-linear confined relation, as expected from previous report [43]. However there was no further confinement of D1R within synapses. Altogether, these data indicate that D1R dynamics is reduced in the postsynaptic area without exhibiting sign of strong confinement degree, as expected if the receptor was engaged in receptor/scaffold proteins interactions, suggesting the presence of another retention mechanism.

### The Interaction between PSD95 and D1R does not Regulate the Receptor Dynamics in Synapses

**Figure 2:**
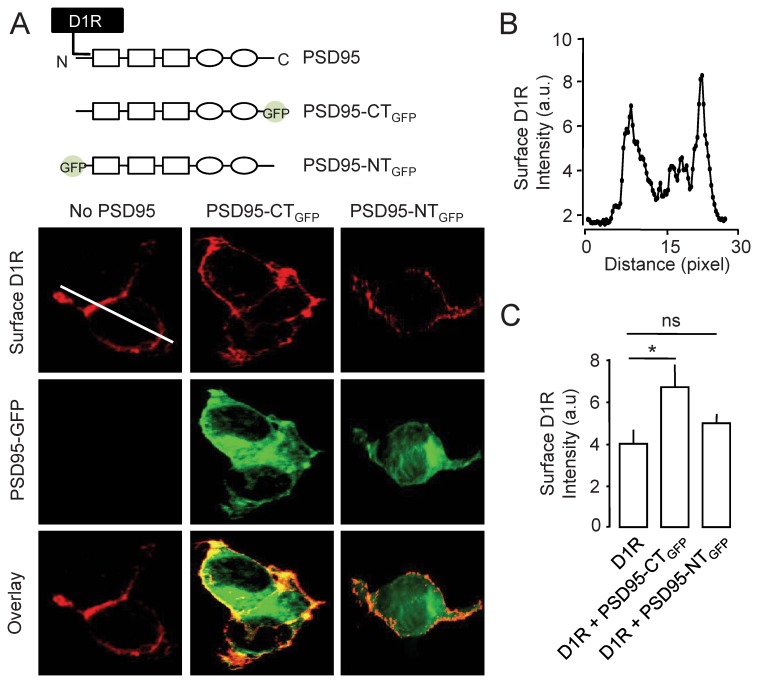
GFP insertion at the N-terminus of PSD-95 prevents the D1R-PSD95 interaction-induced D1R surface delivery. (**A**) Schematic representation of the interaction between PSD-95, characterized by its PDZ binding (squares), SH3 and GK domains (rounds), and D1R (*upper panel*). Note that the interaction occurs at the N-terminus domain of PSD95. Two variants of PSD95 were transfected in HEK cells: PSD95-CT_GFP_ that contains a GFP at its C-terminus and PSD95-NT_GFP_ that contain a GFP at its N-terminus (insertion at amino acid position 32). HEK cells were transfected with D1R-CFP and either PSD95-CT_GFP_ or PSD95-NT_GFP_ (do not bind to D1R). The surface content of D1R was measured by immuncytochemistry in the transfected cells (*lower panel*). (**B**) Line scan (white line in A) of the immunofluorescence of surface D1R from a HEK cell transfected with D1R alone. Note that D1R are enriched at the plasma membrane. (**C**) Quantification of the surface content of D1R in the various conditions. The co-expression of PSD95-CT_GFP_ increases the surface content of D1R (P<0.05), whereas the co-expression of PSD95-NT_GFP_ has no significant effect on the surface content of D1R (P>0.05).

To directly test the presence or absence of a D1R/scaffold protein interaction in the synapse, we generated variants of PSD95 that differentially impact on D1R. Indeed, PSD95 directly interacts with D1R and this interaction regulates the cellular trafficking of D1R by acting on their cycling rate between intracellular and membrane compartments [34], [35] and this interaction is proposed to serve as substrate for the synaptic location of D1R. We developed two variants of PSD95 in which a GFP was inserted either in the C-terminus part (PSD95-CT_GFP_) or at the amino acid 32 in the N-terminus part of PSD95 (PSD95-NT_GFP_). The latter one prevents the interaction of D1R with PSD95 that takes place between amino acids 1 to 46 of the scaffold protein [35]. Since it has been previously reported that the direct binding of PSD95 and D1R favors D1R membrane delivery [35], we measured the effective blockade of the D1R/PSD95 interaction by PSD95-NT_GFP_ in HEK cells expressing D1R either alone or with PSD95-CT_GFP_ or PSD95-NT_GFP_ (Fig. 2A). We thus immunostained surface D1R to quantify the PSD95-induced variations of surface expression profile (Fig. 2A–B). As expected, compared to when D1R expressed alone, the co-expression of PSD95-CT_GFP_ increased the surface delivery of D1R, whereas the co-expression of PSD95-NT_GFP_ did not alter D1R surface content (Fig. 2C), consistent with a lack of direct interaction between D1R and PSD95-NT_GFP_. Beyond the confirmation that the D1R/PSD95 interaction regulates the surface expression of D1R [34], [43], these variants of PSD95 were then used to test the role of the direct interaction between PSD95 and D1R in the synaptic dynamics of the latter one.

**Figure 3:**
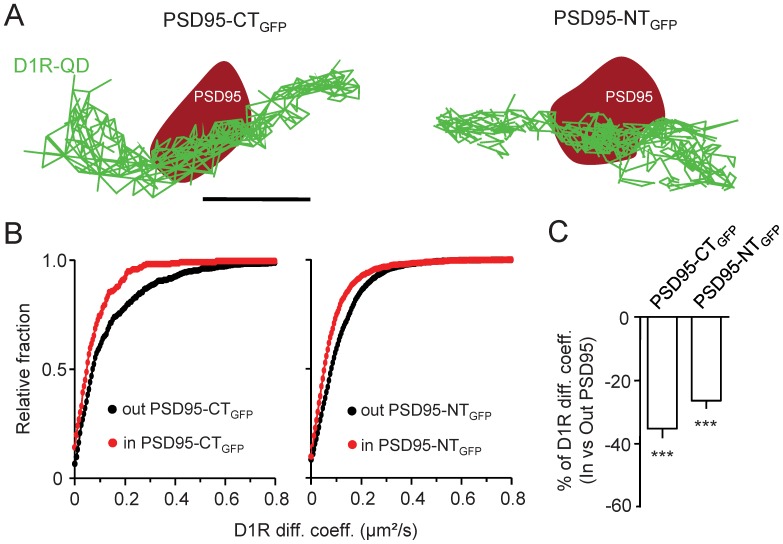
PSD95 content does not regulate D1R surface dynamics in native synaptic structures. (**A**) Representative surface D1R-QD trajectories on hippocampal neurons transfected with either PSD95-CT_GFP_or PSD95-NT_GFP_. Surface D1R-QD (green traces) were tracked in PSD95 synaptic clusters (red areas). Scale bar = 350 nm. (**B**) Frequency distribution of the instantaneous diffusion coefficients of surface D1R either inside or outside of PSD95-CT_GFP_ or PSD95-NT_GFP_ clusters (*left and right panels respectively*). D1R diffusion coefficients tends toward a reduced mobility inside synaptic areas in both conditions (out PSD95-CT_GFP_ n = 546 and in PSD95-CT_GFP_ n = 211; out PSD95-NT_GFP_ n = 2457 and in PSD95-NT_GFP_ n = 1213). (**C**) Comparisons of D1R surface diffusion coefficient variations between outside and inside PSD95-CT_GFP_ or PSD95-NT_GFP_ clusters. Note that the position of the GFP does not affect the slowdown of surface D1R when penetrating a PSD95 cluster, suggesting that D1R surface diffusion is not regulated by PSD95(“Out”: n = 7 neuronal fields, P<0.001; “In”: n = 7 neuronal fields, P<0.001).

Single D1R-QD were tracked in PSD95 clusters of hippocampal neurons expressing either PSD95-NT_GFP_ or PSD95-CT_GFP_ proteins (Fig. 3A).Consistent with the above results, D1R surface diffusion was significantly reduced in the area of PSD95 clusters (Fig. 3B–C), irrespective of the presence of PSD95-NT_GFP_ and PSD95-CT_GFP_ in PSD. Surprisingly, D1R surface trafficking was similarly reduced in PSD95-NT_GFP_and PSD95-CT_GFP_ clusters (Fig. 3B–C), although the presence of PSD95-NT_GFP_ alters the functional interaction between PSD95 and D1R. Taken to phase value these data would indicate that the interaction between the N-terminus part of PSD95 and D1R does not participate into the D1R trapping into glutamate synapses, consistent with the above MSD data. However, a difficulty of these experiments is that the incorporation of the PSD95 variants into native PSD95 clusters might differ, leaving the possibility that the lack of difference observed between the constructs is based on the poor incorporation of, for instance, PSD95-NT_GFP_ into PSD. Additional experiments were thus required to ascertain that the interaction between PSD95 and D1R does not play a direct role in D1R surface dynamics in synapses.

**Figure 4:**
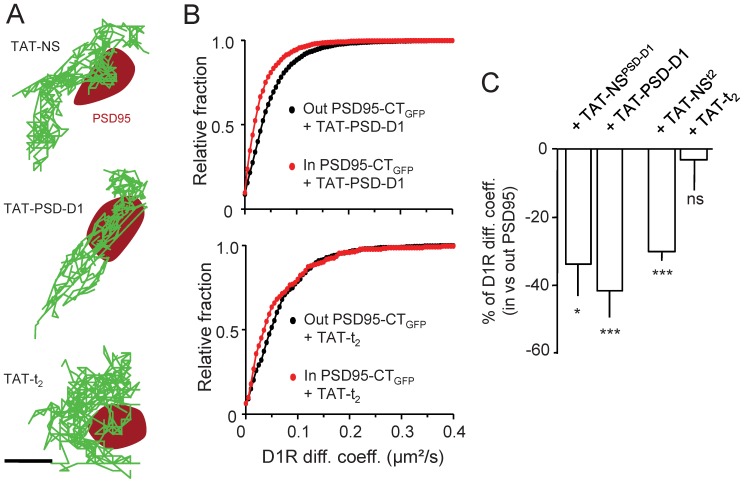
Synaptic D1R surface dynamics are regulated by direct interaction with NMDAR but not with PSD95. (**A**) Representative surface D1R-QD trajectories acquired in presence of either TAT-t_2_ or TAT-PSD-D1 peptide, disrupting the physical interaction of D1R with NMDAR and PSD95 respectively, or their control peptide (respectively TAT-NS^PSD-D1^ and TAT-NS^t2^). Surface D1R-QD (green traces) were tracked on hippocampal neurons transfected with PSD95-GFP (red area). Scale bar = 350 nm. (**B**) Frequency distribution of the instantaneous diffusion coefficients of surface D1R either inside or outside PSD95-GFP clusters in presence of either TAT-PSD-D1 or TAT-t_2_ peptide (*up and down panels respectively*). Surface behavior of D1R was only different when the interaction with NMDAR was prevented by the TAT-t_2_ peptide (out TAT-PSD-D1 n = 4060 and in TAT-PSD-D; n = 1025; out TAT-t_2,_ n = 446 and in TAT-t_2,_ n = 213). (**C**) Comparisons of D1R surface diffusion coefficient variations between outside and inside PSD95-GFP clusters in presence of either TAT-PSD-D1 (TAT-NS^PSD-D1^ 10 µM, 10 min; n = 22 neuronal fields, P>0.05; TAT-PSD-D1, 10 µM, 10 min; n = 14 neuronal fields, P>0.05) or TAT-t_2_ (TAT-NS^t2^, 10 µM, 10 min; n = 6 neuronal fields, P<0.001; TAT-t_2_ 10 µM, 10 min; n = 8 neuronal fields, P>0.05) peptide compared to their respective control non-sense peptide. Note that the slowdown of surface D1R when penetrating a PSD95 cluster, i.e. a postsynaptic density area, was only abolished when the interaction with NMDAR was prevented by the TAT-t_2_ peptide.

To tackle this point we generated a competing peptide,TAT-PSD-D1, which prevents D1R interaction with PSD95 and alter D1R live dynamics as we previously showed [43]. The TAT sequence allows the peptide to penetrate the cells and act at the intracellular level. When neurons were incubated with either TAT-PSD-D1 or a scramble non-sense version of the peptide, TAT-NS^PSD-D1^ (10 µM, 10 min incubation for both peptides), the D1R synaptic dynamics remain unaltered in all conditions (Fig. 4A–C). These data indicate thus that preventing acutely the interaction between endogenous PSD95 and D1R does not impact on the synaptic behavior of D1R. All together, we demonstrate that the interaction between PSD95 and D1R does not play a role in the regulation of D1R surface dynamics and synaptic anchoring in hippocampal neuronal network, leaving open the question of the molecular mechanism responsible for the slowing down of D1R in synapses.

### The Interaction between GluN1-NMDARand D1R Regulates D1R Synaptic Dynamics

Among the potential mechanism, the direct interaction between D1R and the GluN1/2A subunits of NMDAR is of particular interest. Indeed, NMDAR are concentrated in hippocampal glutamate synapses. Most interestingly, the slowdown of synaptic D1R, without a strong confinement, could be explained by the interaction of the receptor with a partner more mobile than scaffold proteins for instance. Since NMDAR diffuse within the postsynaptic area and its vicinity [36], [37] they can thus potentially serve as “weak” anchor for D1R. To directly address this hypothesis, we used a competing peptide, i.e. TAT-t_2_,that prevents the interaction between GluN1 subunit and D1R C-termini [28], [29]. A scramble non-sense version of the peptide, TAT-NS^t2^, was also generated. After incubation of hippocampal neurons with either peptide (10 µM, 10 min incubation), it clearly appeared that TAT-t_2_fully prevented the synaptic retention of D1R, whereas the TAT-NS^t2^ was without any effect (Fig. 4). This indicates that the direct interaction between D1R and NMDAR is required for the dynamic retention of surface D1R in the postsynaptic density area, and that such interaction appears to be responsible for the control of D1R dynamics in the PSD.

## Discussion

We demonstrate in hippocampal neuronal networks that dopamine D1 receptors (D1R) laterally diffuse within glutamate synapses, in which their diffusion is reduced. The disruption of the interaction between D1R and PSD95, using variants of PSD95 or D1R/PSD95 interaction competing peptide, did not affect D1R dynamics in glutamatergic synapses. Strikingly, preventing the physical interaction between D1R and the GluN1 subunit of NMDAR, using competing peptide, fully abolished the synaptic stabilization of diffusing D1R. Together, we report that D1R are dynamically retained in glutamate synapse through a mechanism requiring the interaction of the receptor with NMDAR, shedding new light on the molecular mechanism regulating the synaptic interplay between dopaminergic and glutamatergic signalling.

Over the last decades evidences of functional cross-talk between different neurotransmitter receptor signalling have been described in various brain structures [48]–[50]. The glutamatergic and dopamine cross-talk plays a crucial role in several brain functions, such as motion, reward, and novelty detection [51], [52]. In the hippocampus, the release of dopamine activates postsynaptic membrane D1/5R, which leads to the intracellular activation of PKA, DARPP-32 and other downstream signalling [10]. This signalling cascade can, on the short term range, affect the glutamatergic signalling, through for instance the modulation of glutamate receptor phosphorylation as well as their overall trafficking [4], [5]. On the other hand, the activation of NMDAR in striatal as well as in hippocampal neurons (data not shown) rapidly alter the trafficking of D1R [42]. Thus, the functional interplay between glutamate and dopamine signalling implicates cross-regulation of the receptor function and dynamics. Our study now indicate that the physical interaction between these receptors also participate to this cross-talk since the capacity of a glutamate synapse to retain dopamine receptors, and thus to express dopamine receptor-mediated signalling, is dependent on the dynamic interaction of these receptors. Consistently, the direct interaction between D1R and NMDAR has been recently shown to regulate glutamate synaptic transmission and working memory in rodents [31]. In addition, the activation of D1R reduces the binding to NMDAR [28], which indicates that the above mechanism of synaptic anchoring of D1R by NMDAR is dependent on the level of dopamine and thus subject to fine regulation. It will be of great interest to investigate the role of the dopamine and glutamate release on D1R synaptic anchoring by NMDAR, since this molecular dynamic cross-road could serve as a physiological integrator of the dopamine and glutamate system overall activities.

In the postsynaptic density area, we now provide direct evidence that the direct interaction between PSD95 and D1R [6] does not directly regulate D1R surface behaviour. It has been proposed that the binding of both NMDAR and D1R to PSD95 provided the molecular locus at which dopamine-glutamate transmission cross-talk [34], [35]. In hippocampal glutamate synapses we did not find experimental support for such a claim. It is however possible thatthePSD95/D1R interaction plays a more important role in the D1R membrane cycling, i.e. membrane insertion or endocytosis [34], [35] without directly contributing to the receptor synaptic anchoring. It is important to note that our data do not exclude the possibility that the PSD95/D1R interaction contribute to D1R signalling in glutamate synapses. Indeed, studies in glutamate receptors uncovered that the synaptic content of a given receptor relies both on its content at the plasma membrane, itself dependent on the receptor cycling, and the “trapping” capacity of the synapses to anchor diffusing receptor [53], [54]. It is thus possible that the PSD95/D1R interaction regulates the delivery of D1R in the vicinity of glutamate synapses [5], a process necessary for the subsequent capture of D1R by NMDAR into synapses. Obviously, the regulatory mechanisms that control D1R surface diffusion are yet to be uncovered and further investigations will be necessary to clarify this important issue. Based on recent evidences, it is also possible that the direct interaction between D1R and PSD-95 play a role in both D1R surface dynamics and synaptic retention in striatal neurons [43], with thus neuron-specific molecular interactions to regulate dopamine receptor dynamics.

Finally, direct other interactors of D1R are present at the plasma membrane of hippocampal neurons, including D3R, D2R, adenosine A1 receptor, and N-type calcium channel [55]–[58], providing potential other mechanisms for stabilization in different membrane compartments. D1R interact with intracellular proteins, such as Neurofilament-M and dopamine receptor-interacting protein 78 (DRIP78) [59], [60], that could also serve as intracellular anchors for surface D1R. It is however intriguing that although several of these proteins are present in glutamate synapses, the interaction of D1R with NMDAR appears to mediate most, if not all, the dynamical retention of synaptic D1R. Dissecting the timing and role of these interacting cascades will likely shed key lights on the regulation of D1R trafficking and interplay with the glutamatergic signaling.

In conclusion, the use ofhigh-resolution single nanoparticle imaging provided a unique and powerful way to dissect, at the sub-micron resolution, the intimate behaviour of surface D1R within live glutamate synapses. We demonstrate that the physical interaction between D1R and the GluN1 subunit of NMDAR abolished the synaptic retention of diffusing D1R in hippocampal neuronal networks. These data uncovered that the dopaminergic and glutamatergic interplay already start at the level of receptor dynamics at the plasma membrane, opening new avenues of research on the regulation of the glutamatergic signalling by neuromodulatory system as the dopamine one.
